# Agricultural intensification and cereal aphid–parasitoid–hyperparasitoid food webs: network complexity, temporal variability and parasitism rates

**DOI:** 10.1007/s00442-012-2366-0

**Published:** 2012-05-30

**Authors:** Vesna Gagic, Sebastian Hänke, Carsten Thies, Christoph Scherber, Željko Tomanović, Teja Tscharntke

**Affiliations:** 1 Agroecology, Department of Crop Science, Georg-August-University, Grisebachstrasse 6, 37077 Göttingen, Germany; 2 Institute of Zoology, Faculty of Biology, University of Belgrade, Studentski trg 16, 11000 Belgrade, Serbia

**Keywords:** Community structure, Biodiversity, Biological control, Agroecosystems

## Abstract

Agricultural intensification (AI) is currently a major driver of biodiversity loss and related ecosystem functioning decline. However, spatio-temporal changes in community structure induced by AI, and their relation to ecosystem functioning, remain largely unexplored. Here, we analysed 16 quantitative cereal aphid–parasitoid and parasitoid–hyperparasitoid food webs, replicated four times during the season, under contrasting AI regimes (organic farming in complex landscapes vs. conventional farming in simple landscapes). High AI increased food web complexity but also temporal variability in aphid–parasitoid food webs and in the dominant parasitoid species identity. Enhanced complexity and variability appeared to be controlled bottom-up by changes in aphid dominance structure and evenness. Contrary to the common expectations of positive biodiversity–ecosystem functioning relationships, community complexity (food-web complexity, species richness and evenness) was negatively related to primary parasitism rates. However, this relationship was positive for secondary parasitoids. Despite differences in community structures among different trophic levels, ecosystem services (parasitism rates) and disservices (aphid abundances and hyperparasitism rates) were always higher in fields with low AI. Hence, community structure and ecosystem functioning appear to be differently influenced by AI, and change differently over time and among trophic levels. In conclusion, intensified agriculture can support diverse albeit highly variable parasitoid–host communities, but ecosystem functioning might not be easy to predict from observed changes in community structure and composition.

**Electronic supplementary material:**

The online version of this article (doi:10.1007/s00442-012-2366-0) contains supplementary material, which is available to authorized users.

## Introduction

Agricultural intensification (AI) on a local and a landscape scale is a major cause of biodiversity loss (Foley et al. 2005). Organic farming has been suggested to oppose such changes and to increase components of biodiversity such as species richness (Hole et al. 2005) and evenness (Crowder et al. 2010). Biodiversity may increase and stabilise overall ecosystem function (Tilman et al. 2006), but characteristics of particular species and food web structure are also important factors influencing the response of communities to human-induced habitat loss and alteration (Melian and Bascompte 2002; Sole and Montoya 2006; Brose et al. 2006; Laliberté and Tylianakis 2010). Even when species richness in a trophic guild remains constant, the frequency of their interactions can change greatly due to changes in habitat quality (Tylianakis et al. 2007). The effects of AI on interaction diversity in parasitoid–host food webs have so far been inconclusive, with both negative (Albrecht et al. 2007) and positive (Tylianakis et al. 2007) effects noted, along with unpredictable consequences for ecosystem functioning. Furthermore, most existing studies used pooled long-term samples or a single snapshot in time, and there are still only a few studies with spatio-temporal resolution in food web research across gradients of human impact (de Ruiter et al. 2005; Memmott et al. 2006; Rooney et al. 2008; but see Laliberté and Tylianakis 2010). We address these important questions by investigating the influence of AI on the temporal changes in structure and function of 64 aphid–parasitoid–hyperparasitoid food webs under contrasting levels of AI.

Parasitoids are one of the key agents for controlling agricultural pests (Schmidt et al. 2003; Thies et al. 2005), and together with their hosts and associated host plants, they comprise over half of all known species of multicellular organisms (Hawkins 1994). Hence, revealing the mechanisms that structure host–parasitoid communities is an important task for both basic and applied ecology. Furthermore, the functional significance of the top consumers in this system (i.e. hyperparasitoids) and their spatio-temporal response to land-use intensity may be of particular importance, but remains unknown. It has been shown that higher trophic level organisms often respond more strongly to AI (Kruess and Tscharntke 1994; Holt et al. 1999; Tscharntke et al. 2005), but the consequences of this for food web structure and ecosystem functioning remain largely unpredictable. Spatio-temporal multi-species and multi-trophic approaches may therefore improve our understanding of key ecosystem services such as pest control (Memmott et al. 2006).

Bottom-up control is important for parasitoid food webs (Hawkins 1992; Bukovinszky et al. 2008; Petermann et al. 2010; Scherber et al. 2010). Hence, changes in the host community can also be expected to affect food web interactions. Host communities may be influenced by a number of factors related to AI. Less intensified fields experience fewer disturbances caused by agricultural practices such as fertiliser and pesticide applications (Lampkin et al. 1999), and structurally complex landscapes allow for more host plants per unit area throughout the year. In addition, species-specific effects of nitrogen application on aphid performance (Honek 1991; Duffield et al. 1997; Awmack and Leather 2002; Hambäck et al. 2007) might structure plant-aphid-parasitoid trophic interactions in conventional and organic farms differently.

Here, we analysed aphid–parasitoid–hyperparsitoid community structure in winter wheat fields located in contrasting landscapes with low (organic fields embedded in structurally complex landscapes) versus high (conventional fields embedded in structurally simple landscapes) levels of AI in Germany. Our aim was to select fields that simultaneously vary in the level of AI on the local and landscape scale to maximise contrast in human-induced habitat changes and unravel its influence on ecologically and economically important parasitoid communities. Our study design reflects a situation commonly found in Central European farming systems: organic farms are often situated in areas containing large amounts of semi-natural vegetation; in contrast, conventional farms are mostly found on richer soils and in areas with less semi-natural vegetation (Gibson et al. 2007).

We collected time-series data on aphid–parasitoid and parasitoid–hyperparasitoid food webs, host abundances and parasitism rates at weekly intervals from the period of aphid colonisation to the period of aphid population breakdown (four time periods). We calculated several measures of community complexity, namely food web complexity (quantitative weighted linkage density, interaction diversity, interaction evenness, generality and vulnerability), species richness and evenness. We tested the following hypotheses: (1) a higher AI is related to higher variability in the biodiversity and food web structure over time, because intensive agricultural practices cause greater disturbance to communities; (2) a higher AI allows lower food web complexity, due to the lower species richness and evenness; (3) higher community complexity leads to higher parasitism rates.

## Materials and methods

### The organisms

In Germany, aphid communities (Hemiptera: Sternorrhyncha) in winter wheat fields are dominated by *Sitobion avenae* (Fabricius), *Metopolophium dirhodum* (Walker) and *Rhopalosiphum padi* (Linnaeus), which are attacked by hymenopteran parasitoids belonging to two groups, Aphidiinae (Braconidae, Ichneumonidea) and Aphelinidae (Chalcidoidea) (Adisu et al. 2002). Aphidiinae are primary, solitary endoparasitoids of aphids, with a cosmopolitan distribution, and they represent the largest fraction of the parasitoids infesting aphids (Starý 1988). Primary parasitoid larvae kill aphids by feeding on them internally and forming cocoons (referred to as “mummies”). Primary parasitoids are attacked by secondary parasitoids, and this may disrupt their ability to control aphids (Rosenheim 1998). Secondary parasitoids form two groups, true hyperparasitoids (belonging to the Alloxystinae: Cynipoidea: Charipidae), which feed on a primary larval host in a living aphid, as well as mummy parasitoids [belonging to the Pteromalidae (Chalcidoidea) and Megaspilidae (Ceraphronoidea)], which attack their host in previously mummified aphids (Sullivan and Völkl 1999). Since we are not interested in host use differences between secondary parasitoids here, we will refer to both of these groups as hyperparasitoids. In addition, parasitoid–host dynamics in winter wheat fields may be influenced by predators and pathogens that attack parasitised or unparasitised aphids (Rosenheim 1998), but it was unfeasible to simultaneously quantify these interactions here, and this study is therefore restricted to the parasitoid natural enemy guild (see also Müller et al. 1999).

### Experimental design

The study was carried out in the year 2008 in eight winter wheat fields in the surroundings of Göttingen, Lower Saxony, Germany (see the Electronic supplementary material, ESM, Map S1). We selected fields that simultaneously varied in levels of AI at local (field) and landscape scales (circle with 500 m radius). Four organically managed fields (with no applications of mineral fertiliser and chemical pesticides), embedded in structurally complex landscapes (>30 % were semi-natural habitats) were compared to four conventionally managed fields (with high applications of mineral fertiliser and chemical pesticides), embedded in structurally simple landscapes (>90 % were agricultural habitats). Thus, we had high versus low AI at local and landscape scales (for further details, see Thies et al. 2011). To avoid direct insect mortality, sampling was done on insecticide-free areas in all fields, a 60 m (along the field edge) by 12 m (into the fields) rectangle. Although our focal plots were not directly treated with insecticides, insecticide applications in high-AI fields may destabilise food webs due to the movements of mobile foraging individuals into and out of the treated area, and possible effects of pesticide drift.

### Species examination

Aphids and parasitised aphids (“mummies”) were counted visually on 100 wheat shoots (five randomly chosen subsamples, with 20 shoots on each sampling occasion) per field on a weekly basis starting from wheat flowering in June (after the main period of aphid colonisation of the fields) until wheat peak ripening in July (the period of aphid population breakdown). In addition, we randomly collected ~100 mummies per field at the same time intervals. Altogether, sampling took place over the course of four seven-day periods. All mummies were reared in the laboratory in order to identify the primary and hyperparasitoid species. This allowed us to observe the exact interaction frequencies between aphid and parasitoid species and between parasitoid genera and hyperparasitoid species (assuming no within-genus hyperparasitoid specialisation and no trophic loops, but allowing for fully resolved direct trophic links; see Müller et al. 1999). In primary parasitoid–hyperparasitoid networks, primary parasitoids were identified to the genus level based on mummy morphology (Powell 1982). Hence, species richness and evenness of primary parasitoids was calculated at the species level in aphid–primary parasitoid webs (using only parasitoids that hatched out of aphids) and at the genus level in primary–hyperparasitoid webs (using only genera of parasitoids that were hyperparasitised).

### Network analysis

In total, we analysed 64 quantitative interaction networks, of which 32 were aphid–primary parasitoid and another 32 were primary–hyperparasitoid networks. We calculated quantitative measures of food web complexity, namely linkage density, interaction diversity, generality, vulnerability and interaction evenness (for detailed formulae see Bersier et al. 2002; Tylianakis et al. 2007). Linkage density incorporates generality (the average number of host taxa per parasitoid) and vulnerability (the average number of parasitoid taxa per host), and represents the ratio of the number of trophic interactions to the number of species. Interaction diversity and interaction evenness are analogous to Shannon diversity and evenness, but with trophic interaction instead of species as the base unit.

### Statistical analysis

Data were analysed using the statistical software R 2.11.1 (R Development Core Team 2010). Our experimental design had a total of *N* = 8 landscapes, each repeatedly observed over each of *N* = 4 time intervals (yielding a total *N* = 32). This spatiotemporal structure was accounted for by fitting linear mixed effects models (nlme package, version 3.1-96, Pinheiro and Bates 2000). The fixed-effects part of the models included agricultural intensification (“AI”, two-level factor: low vs. high) and the sampling week (“Week”, numeric, 1–4) as well as interactions between them. Abundances of aphids were not correlated to food web indices, but were highly correlated to AI and Week, so they were not included in the models as a covariate. To account for nonlinearity over time, we used polynomial terms for “Week” when necessary. Fields (“Field”, 1–8) were considered random effects. In *R* notation, the corresponding model structure was *y* ~ AI × Week + I(Week)^2, random = ~1|Field, where *y* is the response variable (parasitism rates, food web structure, species richness, evenness or relative abundance).

We tested for temporal pseudoreplication by inspecting the autocorrelation function (ACF) of the residuals, adjusted for missing values (Zuur et al. 2009). A compound symmetry correlation structure [corCompSymm(form = ~Week)] was used to account for correlations between observations taken at different time points; this assumes an equal correlation of within-group observations across all time points, and is particularly suitable for short time series (Pinheiro and Bates 2000, p. 228). In addition, we used variance functions to model heteroscedasticity when necessary. Models were fitted using restricted maximum likelihood and compared using AICc (Akaike’s information criterion, corrected for small sample sizes). We did not use the Bonferroni or MANOVA approach to correct for multiple testing because adjusting alpha values increases the likelihood of type II error inflation and MANOVA-type approaches decrease in power when the number of tests increases (Moran 2003). This is particularly important for ecological studies, which are often characterised by high variability, a small number of replicates, and consequently low statistical power (Moran 2003; Macfadyen et al. 2009a).

As an estimate of the economic injury level, we calculated the number of aphids per 100 shoots (for a similar approach, see Larsson 2005). To estimate the potential for biological control, we used parasitism rates, and for biological control disruption, hyperparasitism rates. Parasitism rates were calculated as the proportion of parasitised hosts from all hosts, i.e. the number of mummies per 100 shoots/number of aphids per 100 shoots (including mummies) for primary parasitism rates, and the number of emerged hyperparasitoids/all collected mummies (adjusted for density per 100 shoots by calculating the relation of the hyperparasitoids to primary parasitoids in the mummy collection data and applying this ratio to the count data) for hyperparasitism rates.

To test for additional effects of species richness and evenness on the food web metrics and (hyper)parasitism rates, we developed a series of alternative models that included different combinations of these explanatory variables and calculated their Akaike weights (Burnham and Anderson 2002; see ESM Table S4).

To assess the influence of community complexity (species richness, species evenness and food web structure) on (hyper)parasitism rates, we used principal component analysis (PCA). The first three axes of the PCA explained 94 % (PCA1 alone 54 %) of the variation for the aphid–parasitoid indices and 92 % (PCA1 alone 51 %) of the cumulative variation for the primary–hyperparasitoid indices. These PCA axes were then used as explanatory variables for the effects on primary and hyperparasitism rates in linear mixed-effects models (as above).

## Results

### Community composition

**Fig. 1:**
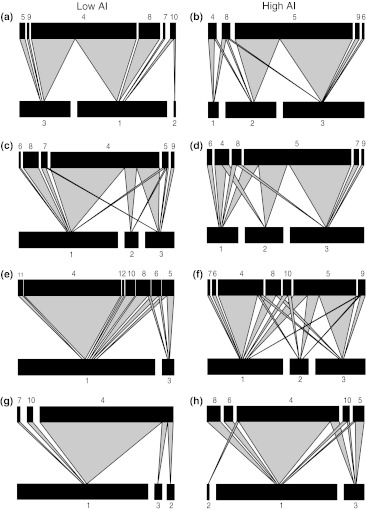
Aphid–parasitoid food webs calculated from pooled data for four fields with low (*left*) and four fields with high (*right*) levels of AI, and in four weekly time series, week 1 (**a** and **b**), week 2 (**c** and **d**), week 3 (**e** and **f**), week 4 (**g** and **h**). *Black bars* represent relative abundances of aphids (*lower bars*) and primary parasitoids (*upper bars*) drawn to different scales. For host and parasitoid densities, see ESM Table S1. The numbers are genera codes from ESM Table S1. Frequency of trophic interactions is indicated by the link width

A total of 1,269 aphid parasitoids emerged from the mummies collected, and 2,311 aphids were counted in the fields, of which 83 % were *S. avenae*, 12 % were *M. dirhodum*, and 5 % were *R. padi*. Aphid, primary and hyperparasitoid communities varied considerably between high- and low-AI fields. as well as over time (Fig. 1; ESM Table S1). Over time, the proportions of parasitised *S. avenae* increased (“Week” *F*_1,21_ = 63.71, *P* < 0.0001), while those of *M. dirhodum* decreased (“Week” *F*_1,21_ = 30.89, *P* < 0.0001) in all fields. Proportions of parasitised *S. avenae* were higher in low-AI fields (“AI” *F*_1,6_ = 13.67, *P* = 0.01), and changed differently over time in fields with contrasting AI regimes (interaction “AI” × “Week^2^” *F*_1,21_ = 5.38, *P* = 0.03), i.e. the response is nonlinear, with a peak at milk-ripening in fields with low AI, while it tends to be more linear and to constantly increase in fields with high AI. The most heavily parasitised aphid in fields with high AI was *M. dirhodum* (“AI” *F*_1,6_ = 5.42, *P* = 0.059), except for the last sampling period. Proportions of *M. dirhodum* and *S. avenae* in fields and in food webs were closely and positively related (*F*_1,20_ = 12.25, *P* = 0.002; *F*_1,20_ = 32.81, *P* < 0.001, respectively).

Proportions of the primary parasitoid *Ephedrus plagiator* in food webs were higher in less intensified fields (“AI” *F*_1,6_ = 33.96, *P* = 0.001), they increased over time (“Week” *F*_1,21_ = 15.37, *P* < 0.001), but they increased faster in fields with high AI (interaction “AI” × “Week”; *F*_1,21_ = 9.11, *P* = 0.006). Proportions of the primary parasitoid *Aphidus rhopalosiphi* were higher in fields with high AI (“AI” *F*_1,6_ = 72.38, *P* = 0.0001), and they decreased nonlinearly over time (“Week^2^” *F*_1,20_ = 5.41, *P* = 0.03), and faster in high-AI fields (interaction “AI” × “Week”; *F*_1,20_ = 22.65, *P* = 0.0001). In the last sampling period (wheat peak ripening), *E. plagiator* dominated in all fields (Fig. 1, ESM Table S1). Proportions of the dominant parasitoids, *Aphidius*, *Ephedrus* and *Praon* in aphid–parasitoid and in parasitoid–hyperparasitoid webs are positively related.

The dominant hyperparasitoid species were *Dendrocerus carpenteri*, *Asaphes suspensus* and *A. vulgaris*. Proportions of *Dendrocerus carpenteri* were higher in fields with low AI (“AI” *F*_1,6_ = 7.71, *P* = 0.03). *A. suspensus* and *A. vulgaris* increased their proportions over time, but showed no response to AI.

### Community complexity: food web indices

**Fig. 2:**
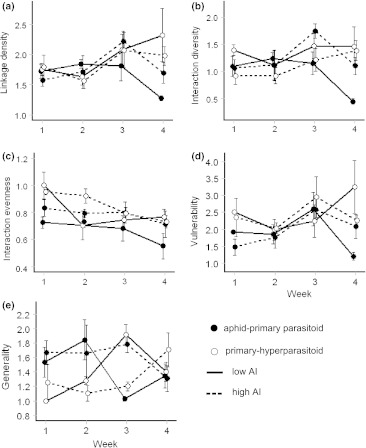
Illustration of aphid–primary parasitoid and primary–hyperparasitoid food web metrics (mean ± SE) across four sampling weeks for low- and high-AI fields

In aphid–primary parasitoid webs, quantitative measures of interaction diversity, interaction evenness, linkage density, generality and vulnerability showed significant changes over time, forming hump-shaped curves with peaks at the time of wheat milk-ripening (week 3) in high-AI fields. We found significant interactions between the level of agricultural intensification and the sampling week for these metrics (Figs. 1 and 2, Table 1; for the mean ± SE, see ESM Table S2).

**Table 1:** *F* values and levels of significance from linear mixed-effects models relating food web metrics (linkage density, interaction diversity, interaction evenness, vulnerability and generality), (hyper)parasitism rates and aphid density for aphid–primary parasitoid webs and primary–hyperparasitoid webs to two predictive factors: (1) agricultural intensification and (2) sampling week (including polynomial terms for “Week”)

	AI	Week	Week^2^	Week^3^	AI:Week	AI: Week^*P*^
Aphid–primary parasitoid						
Linkage density	NS	23.42***	14.20**	NS	6.52*	NS
Interaction diversity	10.38*	32.05***	18.19**	NS	7.52*	NS
Interaction evenness	NS	4.35*	NS	NS	NS	NS
Vulnerability	NS	NS	8.88***	6.48*	7.86**	NS
Generality	NS	4.49*	NS	NS	NS	5.31*
Primary parasitism rate	NS	9.82**	NS	NS	5.88*	NS
Primary–hyperparasitoid						
Linkage density	NS	NS	NS	NS	NS	NS
Interaction diversity	NS	7.17*	NS	NS	NS	NS
Interaction evenness	NS	45.76***	NS	NS	NS	NS
Vulnerability	NS	NS	NS	NS	NS	NS
Generality	NS	8.69**	NS	NS	NS	13.63**
Hyperparasitism rate	NS	53.82***	NS	NS	4.60*	NS
Aphid density	NS	NS	NS	5.41*	5.67*	NS

A strict interpretation (corrected for multiple testing) would render only *P* values <0.003 significant (but see “Materials and methods” section for arguments against correcting for multiple testing)

* *p* < 0.05; ** *p* < 0.01; *** *p* < 0.001; *NS p* > 0.05

^*P*^Polynomial, i.e. 2 or 3

In primary parasitoid–hyperparasitoid webs, quantitative values of interaction evenness, interaction diversity and generality changed significantly over time (Fig. 2; Table 1; ESM Table S2). Generality increased faster over time in low-AI fields and formed a hump-shaped curve at wheat milk-ripening. Interaction diversity increased over time, while interaction evenness decreased.

### Community complexity: species richness and evenness

Species richness and evenness of different trophic level organisms changed differently over time and between low- and high-AI fields (ESM Fig. S1). Species richness of aphids increased faster in fields with low AI over time (interaction “AI” × “Week^3^”, *F*_1,17_ = 4.95, *P* = 0.039), while evenness of aphids changed over time (“Week” *F*_1,22_ = 5.60, *P* = 0.02), with a trend for higher evenness values in fields with high AI (“AI” *F*_1,6_ = 4.89, *P* = 0.06), and the highest values obtained at milk-ripening in these fields. Primary parasitoid species richness and evenness changed nonlinearly over time (“Week^3^” *F*_1,19_ = 9.78, *P* = 0.005; *F*_1,20_ = 9.16, *P* = 0.007, respectively), were highest at wheat milk-ripening in all fields, and remained high at the end of the sampling season only in fields with high AI levels (interaction “AI” × “Week”, *F*_1,19_ = 8.25, *P* = 0.009; *F*_1,19_ = 8.90, *P* = 0.007, respectively).

In primary–hyperparasitoid webs, species richness and evenness of primary parasitoids and hyperparasitoids increased over time in all fields (“Week” *F*_1,18_ = 20.71, *P* < 0.001; *F*_1,19_ = 9.57, *P* = 0.006; *F*_1,20_ = 10.81, *P* = 0.004; *F*_1,22_ = 4.15, *P* = 0.054, respectively), and hyperparasitoid species richness had (with marginal significance) higher values in fields with low AI (“AI” *F*_1,6_ = 4.35, *P* = 0.08). Richness and evenness of primary parasitoids in primary–hyperparasitoid webs reached a maximum at milk-ripening, but only in low-AI fields (interaction: AI × Week^2^, *F*_1,19_ = 11.52, *P* = 0.003; *F*_1,19_ = 8.68, *P* = 0.008).

### Ecosystem function: aphid abundances, parasitism and hyperparasitism rates

**Fig. 3:**
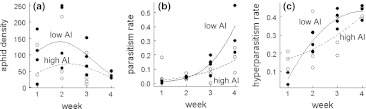
Model predictions for primary parasitism rates (**a**), hyperparasitism rates (**b**), and aphid density (**c**) across four weeks in low-AI fields (*filled line*) and high-AI fields (*dashed line*)

Aphid abundances formed hump-shaped curves, with the highest peak occurring in the second sampling period in low-AI fields. Primary and hyperparasitism rates increased over time, and increased faster in fields with low AI (Table 1; Fig. 3; for the mean ± SE, see ESM Table S2).

### Biodiversity–ecosystem functioning relationship

Primary parasitism rate was negatively related to the first two PCA axes (PCA1, *F*_1,18_ = 5.53, *P* = 0.03; PCA2, *F*_1,18_ = 6.61, *P* = 0.01; see ESM Table S3 for the description of PCAs). Hyperparasitism rate was positively related to the first axis (PCA1, *F*_1,14_ = 75.36, *P* < 0.001) and negatively related to the second and third axes (PCA2, *F*_1,14_ = 29.18, *P* < 0.001; PCA3, *F*_1,14_ = 37.69, *P* < 0.001). All community complexity variables (i.e. food web indices and species richness and evenness) were positively related to PCA1. Hence, our results indicate an overall negative relation between primary parasitism rates and community complexity, but an overall positive relation between hyperparasitism rates and community complexity.

## Discussion

In this study, we found distinct differences in aphid, parasitoid and hyperparasitoid communities between fields with low and high AI and over time. Aphid–parasitoid diversity and food web structure showed greater changes over time in fields with high AI, higher food web complexity, but lower parasitism rates. Highly intensified fields were mainly colonised by leaf-colonising aphids (*M. dirhodum, R. padi*), which may have benefited from the higher nitrogen levels (Honek 1991; Hasken and Poehling 1995) that arise from the high amounts of inorganic fertilisers applied in conventionally managed fields. This may be due to the greater amounts of amino acids in the phloem sap of treated plants, increased leaf area, leaf chlorophyll content and/or the number of shoots per plant of treated compared to untreated plants (Honek 1991; Riedell and Kieckhefer 1993; Hasken and Poehling 1995; Duffield et al. 1997). On the other hand, the dominant aphid species in fields with low AI, *S. avenae,* has been shown to be less influenced by nitrogen (Honek 1991; Hasken and Poehling 1995). It benefits from a higher percentage of grassland in structurally complex landscapes (Schmidt et al. 2004; Purtauf et al. 2005), which serve as hibernating sites (Leather 1993; Thies et al. 2005). These differences in aphid communities appear to have induced bottom-up effects of changes in primary and hyperparasitoid community composition and food web structure. The identity of the dominant primary parasitoid species differed between fields with high (*A. rhopalosiphi*, commonly associated with *M. dirhodum* and *R. padi*) and low (*E. plagiator*, commonly associated with *S. avenae*) AI at the time of aphid colonisation. This should have large implications for biological control (given differences in the dominant parasitoid species identity between fields with contrasting AI regimes but similar total parasitism rates at wheat flowering), because parasitoids that are active early in the year are important for maintaining aphid densities at low levels (Langer et al. 1997). The identity of the dominant parasitoid species also changed over time within fields with high AI, as leaf nutritional quality decreased and the proportions of ear-colonising aphid *S. avenae* increased, with possible influences on the parasitoid species pool in the next year. In addition, the dominant hyperparasitoid species in low-AI fields, *D. carpenteri*, increased as *E. plagiator* and *S. avenae* proportions increased, whereas in fields with high AI, *A. suspensus* and *A. vulgaris* dominated. These results emphasise the changing identities of the one or few species that dominate communities and ecosystem processes. Changes in the dominance structure under the influence of AI suggest that management strategies should be adapted to different key species and AI levels, for example favouring specific alternative host species that would support different parasitoids in different landscapes. However, dominance structure may change among years and regions, and long-term studies are needed before recommendations of adjusted management strategies are possible.

Changes in aphid–parasitoid network complexity (linkage density, interaction diversity, generality and vulnerability) under different AI regimes, with more distinct nonlinear changes in fields with high AI over time, were best explained by models that included evenness of both trophic levels. Evenness of aphids, showed similar changes to those in food web metrics, increased faster over time in fields with high AI, and formed hump-shaped curves, reaching their peaks at the milk-ripening period (the period of aphid reproduction in fields). Primary parasitoid species richness and evenness in aphid–parasitoid webs were highest in the milk-ripening period in all fields, and remained high in fields with high AI. This is contrary to findings by Crowder et al. (2010), who found organic farming to promote predator evenness. The nonlinearity in food web descriptors and higher aphid–parasitoid network complexity in our study did not simply result from higher aphid and parasitoid abundances, as they increased faster over time in fields with low levels of AI. However, complexity of biotic interactions can also decrease as species abundances decrease (Albrecht et al. 2007; Tylianakis et al. 2007). Our results support findings obtained by Gagic et al. (2011), who found aphid–parasitoid food web complexity to increase with landscape structural simplification. However, their study was a snapshot in time, conducted at wheat milk-ripening, and missed temporal changes in food web structure. In primary–hyperparasitoid webs, generality was higher in fields with low AI, reaching a peak at wheat milk-ripening, and the best model for generality included evenness of the lower trophic level that followed the same pattern.

Parasitism and hyperparasitism rates were higher in fields with low-intensity agriculture, presumably due to the higher availability of alternative resources in structurally complex landscapes. There is evidence that organic farming has no or only little influence on parasitoid abundances (Roschewitz et al. 2005; Macfadyen et al. 2009b), whereas landscape simplification can decrease parasitoid abundances (Thies et al. 2005; Roschewitz et al. 2005), resulting in lower biological control. Increases in parasitism rates over time appeared to be due to increases in parasitoid total abundances, rather than to changes in species identity (indicating a certain degree of functional redundancy or temporal complementarity among these species), given greater increases in parasitism rates, but smaller changes in parasitoid dominance structure over time in fields with low AI compared to fields with high AI. Moreover, we are not aware of any published evidence that species which are dominant later in the season in all fields (*E. plagiator*) are more efficient than other parasitoids, while, in contrast, *A. rhopalosiphi* is often reported to be one of the most efficient parasitoids of cereal aphids (Farrell and Stufkens 1990; Levie et al. 2005; Adisu et al. 2002). Hyperparasitism rates were better explained by models including species richness than species evenness. However, when analysing this together with food web metrics in multivariate analysis, there was no single best predictor of (hyper)parasitism rates. More generally, parasitism rates were negatively related to the community complexity indices, supporting findings that parasitoids function better in simplified food webs dominated by a single link (Hawkins et al. 1999; Montoya et al. 2003; Finke and Denno 2004; Tylianakis et al. 2007). In contrast, hyperparasitism rates were positively related to overall community complexity in our study, supporting the traditional view of a positive biodiversity–ecosystem functioning relationship.

In conclusion, aphid–parasitoid–hyperparasitoid community structure markedly changed under different AI regimes. Over time, changes in the identity of the dominant species and increases in community variability (nonlinear increases in aphid–parasitoid food web complexity) in high-AI fields were presumably due to the bottom-up effect of plant nutritional quality, more specifically nitrogen availability. Despite similar food web structures and species richnesses at the time of aphid colonisation, the identities of the dominant parasitoid species differed between fields with high and low AI, indicating the importance of focusing on both species- and community-level analysis to understand ecosystem functioning. Aphid–parasitoid community complexity was negatively related to parasitism rates, thus contradicting common expectations of a positive biodiversity–ecosystem functioning relationship. Thus, intensified agricultural fields may support a diverse but highly variable parasitoid–host community, but ecosystem functioning may not be easy to predict based on observed changes in community structure and composition.

## Electronic supplementary material

Below is the link to the electronic supplementary material.

Supplementary material 1 (DOC 77 kb)

Supplementary material 2 (DOC 63 kb)

Supplementary material 3 (DOC 45 kb)

Supplementary material 4 (DOC 52 kb)

Supplementary material 5 (TIFF 5556 kb)

Supplementary material 6 (EPS 1624 kb)
