# Supplementary material for: Agricultural intensification and cereal aphid–parasitoid–hyperparasitoid food webs: network complexity, temporal variability and parasitism rates
Source: Oecologia. 2012 May 30;170(4):1099–109. doi: 10.1007/s00442-012-2366-0 (PMC3496544; doi:10.1007/s00442-012-2366-0)
Supplement: Supplementary file 1 — Supplementary material 1 (DOC 77 kb) [file 442_2012_2366_MOESM1_ESM.doc]

Electronic Supplementary Materials for *Oecologia*

**Agricultural intensification and cereal aphid-parasitoid-hyperparasitoid food webs: network complexity, temporal variability and parasitism rates**

^1^ Agroecology, Department of Crop Science, Georg-August-University, Grisebachstrasse 6, 37077 Göttingen, Germany

^2^ Institute of Zoology, Faculty of Biology, University of Belgrade, Studentski trg 16, 11000, Belgrade, Serbia

** Correspondence:* Phone +49 551 3922157, Fax +49 551 398806, E-mail: [vgagic@gwdg.de](mailto:vgagic@gwdg.de)

**ESM Table 1*.*** Arithmetic means ± standard errors of abundances of aphids, primary and hyperparasitoid species per 100 shoots in high and low AI fields and across four weeks

|  | High AI | | | |  | Low AI | | | | |
| --- | --- | --- | --- | --- | --- | --- | --- | --- | --- | --- |
| Taxa code | 1 | 2 | 3 | 4 |  | 1 | 2 | 3 | | 4 |
| Aphids |  |  |  |  |  |  |  |  |  | |
| *1* *S. avenae* | 27.50±23.8 | 57.00±36.8 | 24.75±16.5 | 32.33±5.36 |  | 86.75±34.8 | 152.25± 44.52 | 79.2±21.2 | 37.67±6.12 | |
| *2*  *R. padi* | 2.25±1.31 | 5.50±2.72 | 3.50± 2.60 | 3.33±2.85 |  | 3.00±1.58 | 4.50±3.84 | 4.25±1.44 | 0.00±0.00 | |
| *3*  *M. dirhodum* | 17.25±8.10 | 17.00±6.42 | 12.25±4.00 | 10.00±9.02 |  | 6.75±3.09 | 7.50±3.52 | 3.75±3.09 | 0.00±0.00 | |
| Primary parasitoids |  |  |  |  |  |  |  |  |  | |
| *4* *E. plagiator* | 0.23±0.23 | 0.27± 0.23 | 0.92± 0.34 | 6.42± 2.18 |  | 1.27±0.64 | 5.15±1.30 | 6.32±1.58 | 26.63±16.6 | |
| *5* *A. rhopalosiphi* | 1.39±0.64 | 2.32±0.64 | 1.96±1.23 | 0.76±0.21 |  | 0.02±0.02 | 0.36±0.18 | 1.31±0.62 | 0.00±0.00 | |
| *6* *A. ervi* | 0.00±0.00 | 0.15±0.09 | 0.10±0.08 | 1.01±0.46 |  | 0.03±0.03 | 0.06±0.06 | 1.25±0.65 | 0.00±0.00 | |
| *7* *A. uzbekistanicus* | 0.00±0.00 | 0.13±0.08 | 0.01±0.01 | 0.00±0.00 |  | 0.02±0.02 | 0.36±0.21 | 0.00±0.00 | 0.13±0.13 | |
| *8* *A. avenae* | 0.00±0.00 | 0.00±0.00 | 0.00±0.00 | 0.00±0.00 |  | 0.00±0.00 | 0.00±0.00 | 0.42±0.42 | 0.00±0.00 | |
| *9* *P. volucrae* | 0.04±0.04 | 0.15±0.09 | 0.58±0.34 | 1.71±1.29 |  | 0.38±0.35 | 0.97±0.55 | 3.48±2.71 | 0.00±0.00 | |
| *10 P. gallicum* | 0.08±0.08 | 0.08±0.08 | 0.14±0.14 | 0.00±0.00 |  | 0.00±0.00 | 0.14±0.10 | 0.00±0.00 | 0.00±0.00 | |
| *11 P. abjectum* | 0.00±0.00 | 0.00±0.00 | 0.00±0.00 | 0.00±0.00 |  | 0.00±0.00 | 0.72±0.72 | 0.00±0.00 | 0.00±0.00 | |
| *12 A. abdominalis* | 0.00±0.00 | 0.00±0.00 | 0.29±0.15 | 0.41±0.33 |  | 0.07± 0.08 | 0.00±0.00 | 0.95±0.66 | 0.57±0.40 | |
| Hyperparasitoids |  |  |  |  |  |  |  |  |  | |
| *13* *D. carpenteri* | 0.12±0.12 | 0.10±0.11 | 0.27±0.17 | 0.77±0.62 |  | 0.04±0.02 | 2.04±0.91 | 6.60±2.96 | 7.01±2.31 | |
| *14* *P. villosa* | 0.00±0.00 | 0.08±0.05 | 0.00±0.00 | 0.30±0.30 |  | 0.00±0.00 | 0.00±0.00 | 0.12±0.07 | 0.66±0.27 | |
| *15* *P. muscarum* | 0.00±0.00 | 0.00±0.00 | 0.00±0.00 | 0.00±0.00 |  | 0.00±0.00 | 0.00±0.00 | 0.07±0.07 | 0.79±0.64 | |
| *16* *P. aphidis* | 0.00±0.00 | 0.00±0.00 | 0.00±0.00 | 0.00±0.00 |  | 0.00±0.00 | 0.00±0.00 | 0.00±0.00 | 0.34±0.34 | |
| *17* *C. clavata* | 0.00±0.00 | 0.00±0.00 | 0.03±0.03 | 0.00±0.00 |  | 0.00±0.00 | 0.00±0.00 | 0.00±0.00 | 0.43±0.31 | |
| *18* *A. brevis* | 0.00±0.00 | 0.02±0.02 | 0.03±0.03 | 0.00±0.00 |  | 0.02±0.02 | 0.23±0.10 | 0.29±0.08 | 1.56±1.30 | |
| *19* *A. victrix* | 0.09±0.06 | 0.27±0.10 | 0.25±0.15 | 0.21±0.10 |  | 0.02±0.02 | 0.07±0.04 | 0.25±0.25 | 0.52±0.30 | |
| *20 A. suspensus* | 0.05±0.03 | 0.40±0.25 | 0.45±0.30 | 2.19±0.94 |  | 0.02±0.02 | 0.15±0.08 | 0.99±0.61 | 5.05±4.20 | |
| *21 A. vulgaris* | 0.03±0.03 | 0.07±0.07 | 0.62±0.40 | 2.92±2.24 |  | 0.00±0.00 | 0.15±0.08 | 0.99±0.68 | 6.13±5.21 | |
