# Supplementary material for: Agricultural intensification and cereal aphid–parasitoid–hyperparasitoid food webs: network complexity, temporal variability and parasitism rates
Source: Oecologia. 2012 May 30;170(4):1099–109. doi: 10.1007/s00442-012-2366-0 (PMC3496544; doi:10.1007/s00442-012-2366-0)
Supplement: Supplementary file 2 — Supplementary material 2 (DOC 63 kb) [file 442_2012_2366_MOESM2_ESM.doc]

Electronic Supplementary Materials for *Oecologia*

**Agricultural intensification and cereal aphid-parasitoid-hyperparasitoid food webs: network complexity, temporal variability and parasitism rates**

^1^ Agroecology, Department of Crop Science, Georg-August-University, Grisebachstrasse 6, 37077 Göttingen, Germany

^2^ Institute of Zoology, Faculty of Biology, University of Belgrade, Studentski trg 16, 11000, Belgrade, Serbia

** Correspondence:* Phone +49 551 3922157, Fax +49 551 398806, E-mail: [vgagic@gwdg.de](mailto:vgagic@gwdg.de)

**ESM Table 2.** Arithmetic means ± standard errors of quantitative measures of interaction diversity, linkage density, interaction evenness, generality, vulnerability, primary and hyperparasitism rates and aphid density in high and low AI fields and across four weeks.

|  |  | |  | | Week | | | |  | |
| --- | --- | --- | --- | --- | --- | --- | --- | --- | --- | --- |
|  |  | |  | | 1 | 2 | 3 | 4 |  | |
| Aphid-primary parasitoid food-webs | | | | |  |  |  |  |  | |
|  | Interaction diversity | | | HighAI | 1.06±0.15 | 1.11±0.28 | 1.75±0.13 | 1.10± 0.16 |  | |
|  |  |  |  | LowAI | 1.09±0.19 | 1.24±0.10 | 1.15±0.21 | 0.44±0.05 |  | |
|  | Linkage density | | | HighAI | 1.57±0.09 | 1.71±0.29 | 2.22±0.17 | 1.69±0.17 |  | |
|  |  |  |  | LowAI | 1.73±0.12 | 1.85±0.07 | 1.81±0.24 | 1.27±0.04 |  | |
|  | Interaction evenness | | | HighAI | 0.83±0.06 | 0.79±0.03 | 0.80±0.08 | 0.71±0.10 |  | |
|  |  |  |  | LowAI | 0.72±0.04 | 0.73±0.04 | 0.68±0.09 | 0.55±0.09 |  | |
|  | Generality | | | HighAI | 1.67±0.16 | 1.66±0.39 | 1.79±0.12 | 1.31±0.18 |  | |
|  |  |  |  | LowAI | 1.54±0.21 | 1.85±0.28 | 1.03±0.26 | 1.35±0.16 |  | |
|  | Vulnerability | | | HighAI | 1.47±0.23 | 1.76±0.32 | 2.57±0.32 | 2.08±0.35 |  | |
|  |  |  |  | LowAI | 1.92±0.03 | 1.85±0.28 | 2.60±0.49 | 1.20±0.12 |  | |
| Parasitism rate | | | | HighAI | 0.06±0.04 | 0.05±0.01 | 0.08±0.03 | 0.17±0.05 |  | |
|  |  |  |  | LowAI | 0.01±0.01 | 0.04±0.003 | 0.13±0.03 | 0.36±0.10 |  | |
| Primary-hyperparasitoid food webs | | | | |  |  |  |  |  |  |
|  | | Interaction diversity | | HighAI | 0.92±0.15 | 0.92±0.15 | 1.22±0.21 | 1.38±0.18 |  | |
|  |  |  |  | LowAI | 1.39±0.10 | 1.11±0.27 | 1.47±0.24 | 1.46±0.36 |  | |
|  | | Linkage density | | HighAI | 1.80±0.20 | 1.56±0.13 | 2.07±0.30 | 1.98±0.15 |  | |
|  |  |  |  | LowAI | 1.75±0.10 | 1.63±0.14 | 2.08±0.27 | 2.32±0.44 |  | |
|  | | Interaction evenness | | HighAI | 0.95±0.02 | 0.92±0.05 | 0.79±0.04 | 0.73±0.05 |  | |
|  | |  |  | LowAI | 1.00±0.01 | 0.70±0.10 | 0.74±0.07 | 0.77±0.06 |  | |
|  | | Generality | | HighAI | 1.25±0.25 | 1.10±0.10 | 1.20±0.06 | 1.71±0.24 |  | |
|  | |  |  | LowAI | 1.00±0.10 | 1.28±0.06 | 1.92±0.15 | 1.41± 0.13 |  | |
|  | | Vulnerability | | HighAI | 2.34±0.57 | 2.02±0.16 | 2.95± 0.60 | 2.25± 0.17 |  | |
|  | |  |  | LowAI | 2.50±0.01 | 1.98±0.30 | 2.24±0.48 | 3.24±0.78 |  | |
| Hyperparasitism rate | | | | HighAI | 0.16±0.03 | 0.23±0.07 | 0.28±0.06 | 0.42±0.02 |  | |
|  |  |  |  | LowAI | 0.06±0.03 | 0.27±0.03 | 0.39±0.02 | 0.43±0.02 |  | |
| Aphid density | | | | HighAI | 47.0±28.2 | 79.5±45.8 | 40.5±21.8 | 45.7±5.33 |  | |
|  | | | | LowAI | 96.5±34.6 | 164.5±48.5 | 87.2±24.0 | 37.7±6.12 |  | |
|  |  | | |  |  |  |  |  |  | |
