# Supplementary material for: Agricultural intensification and cereal aphid–parasitoid–hyperparasitoid food webs: network complexity, temporal variability and parasitism rates
Source: Oecologia. 2012 May 30;170(4):1099–109. doi: 10.1007/s00442-012-2366-0 (PMC3496544; doi:10.1007/s00442-012-2366-0)
Supplement: Supplementary file 3 — Supplementary material 3 (DOC 45 kb) [file 442_2012_2366_MOESM3_ESM.doc]

Electronic Supplementary Materials for *Oecologia*

**Agricultural intensification and cereal aphid-parasitoid-hyperparasitoid food webs: network complexity, temporal variability and parasitism rates**

^1^ Agroecology, Department of Crop Science, Georg-August-University, Grisebachstrasse 6, 37077 Göttingen, Germany

^2^ Institute of Zoology, Faculty of Biology, University of Belgrade, Studentski trg 16, 11000, Belgrade, Serbia

** Correspondence:* Phone +49 551 3922157, Fax +49 551 398806, E-mail: [vgagic@gwdg.de](mailto:vgagic@gwdg.de)

**ESM Table 3.** Principal component analysis

Correlation table for the first three principal component axes with (a) aphid-primary parasitoid and (b) primary-hyperparasitoid community complexity indices.

(a)

| Aphid-primary parasitoid webs | PC1 | PC2 | PC3 |
| --- | --- | --- | --- |
| Generality | 0.249009 | 0.46927 | -0.05197 |
| Linkage density | 0.415581 | -0.11161 | -0.15655 |
| Interaction evenness | 0.301532 | 0.13611 | -0.71458 |
| Interaction diversity | 0.446175 | -0.01574 | 0.03911 |
| Vulnerability | 0.294561 | -0.43702 | -0.17884 |
| Higher trophic level evenness | 0.332730 | -0.37260 | 0.06305 |
| Lower trophic level evenness | 0.306636 | 0.42865 | 0.10673 |
| Higher trophic level richness | 0.319295 | -0.29329 | 0.50635 |
| Lower trophic level richness | 0.286082 | 0.38597 | 0.39610 |

(b)

| Primary-hyperparasitoid webs | PC1 | PC2 | PC3 |
| --- | --- | --- | --- |
| Generality | 0.201399 | -0.50710 | 0.06862 |
| Linkage density | 0.425232 | 0.10889 | -0.08592 |
| Interaction evenness | 0.077185 | 0.24275 | 0.90233 |
| Interaction diversity | 0.452479 | -0.00861 | 0.07450 |
| Vulnerability | 0.375503 | 0.32793 | -0.12122 |
| Higher trophic level evenness | 0.352128 | 0.30731 | 0.03931 |
| Lower trophic level evenness | 0.265323 | -0.45148 | 0.27178 |
| Higher trophic level richness | 0.416592 | 0.12272 | -0.27863 |
| Lower trophic level richness | 0.242857 | -0.50111 | -0.02059 |
