# Supplementary material for: Agricultural intensification and cereal aphid–parasitoid–hyperparasitoid food webs: network complexity, temporal variability and parasitism rates
Source: Oecologia. 2012 May 30;170(4):1099–109. doi: 10.1007/s00442-012-2366-0 (PMC3496544; doi:10.1007/s00442-012-2366-0)
Supplement: Supplementary file 4 — Supplementary material 4 (DOC 52 kb) [file 442_2012_2366_MOESM4_ESM.doc]

Electronic Supplementary Materials for *Oecologia*

**Agricultural intensification and cereal aphid-parasitoid-hyperparasitoid food webs: network complexity, temporal variability and parasitism rates**

^1^ Agroecology, Department of Crop Science, Georg-August-University, Grisebachstrasse 6, 37077 Göttingen, Germany

^2^ Institute of Zoology, Faculty of Biology, University of Belgrade, Studentski trg 16, 11000, Belgrade, Serbia

** Correspondence:* Phone +49 551 3922157, Fax +49 551 398806, E-mail: [vgagic@gwdg.de](mailto:vgagic@gwdg.de)

**ESM Table 4.** We used Akaike weights to test for for additional effects of species richness and evenness on the food web metrics. The series of alternative linear mixed-effects models were developed in the same manner, as in the main analysis (see Material and methods section in the main document). Since “AI” and “Week” are design-based variables and there was good evidence that they were important for the explanation of variation in our response variables (see results), we included them in all models (for justification see Zuur et al. 2009 Mixed effects models and extensions in ecology with R. Statistics for biology and health. Springer, New York, pp. 483-485). These models can be thought of as different ‘hypotheses’ about the relationships between response and explanatory variables. We fitted models without interactions and used variance-inflation factors (VIF) to assess the extent of collinearity. The collinearity was low, and in all cases VIFs were well below 10 (cf. Zuur et al. 2009). We then calculated the relative probability of each model being the best model by calculating their Akaike weights, using AICc (Burnham and Anderson 2002 Model selection and multimodel inference: a practical information-theoretic approach. 2nd edn. Springer-Verlag, New York). The model rankings and Akaike weights (*wi*) for the best three models relating food web metrics and (hyper-) parasitism rates to (1) Agricultural intensification, “1”, (2) Sampling Week “2”, (3) lower trophic level evenness “3”, (4) higher trophic level evenness “4”, (5) higher trophic level richness “5”, (6) lower trophic level richness “6” are presented in the table. In aphid-primary parasitoid webs, models that included evenness of aphids and parasitoids had the highest relative probability of being the best models (except for interaction evenness, which included also aphid species richness). In primary-hyperparasitoid webs, the best models for the explanation of interaction diversity and vulnerability included evenness of both trophic levels and richness of hyperparasitoids, and for interaction evenness, evenness of both trophic levels and the richness of primary parasitoids. The best models for the explanation of linkage density and generality included hyperparasitoid richness and primary parasitoid evenness, respectively.

|  | Model 1 (*wi*) | Model 2 (*wi*) | Model 3 (*wi*) |  |
| --- | --- | --- | --- | --- |
| Aphid-primary parasitoid | | | |  |
| Linkage density | 3+4 (0.63) | 4 (0.20) | 4+6 (0.06) |  |
| Interaction diversity | 3+4 (0.92) | 3+4+6(0.04) | 4 (0.02) |  |
| Interaction evenness | 3+6 (0.48) | 3+4+6 (0.25) | 3 (0.09) |  |
| Vulnerability | 4 (0.60) | 3+4 (0.21) | 4+6 (0.14) |  |
| Generality | 3+4 (0.56) | 3 (0.33) | 3+6 (0.05) |  |
| Primary-hyperparasitoid | | | |  |
| Linkage density | 5 (0.74) | 4+5 (0.13) | 3+5 (0.08) |  |
| Interaction diversity | 3+5 (0.43) | 3+4+5 (0.43) | 5+6 (0.05) |  |
| Interaction evenness | 4 (0.42) | 3+4+6 (0.35) | 3+4 (0.20) |  |
| Vulnerability | 3+5 (0.40) | 3+4+5 (0.32) | 4+5 (0.11) |  |
| Generality | 3 (0.80) | 3+4 (0.12) | 3+6 (0.04) |  |
