# Supplementary figures and images for: Agricultural intensification and cereal aphid–parasitoid–hyperparasitoid food webs: network complexity, temporal variability and parasitism rates
Source: Oecologia. 2012 May 30;170(4):1099–109. doi: 10.1007/s00442-012-2366-0 (PMC3496544; doi:10.1007/s00442-012-2366-0)

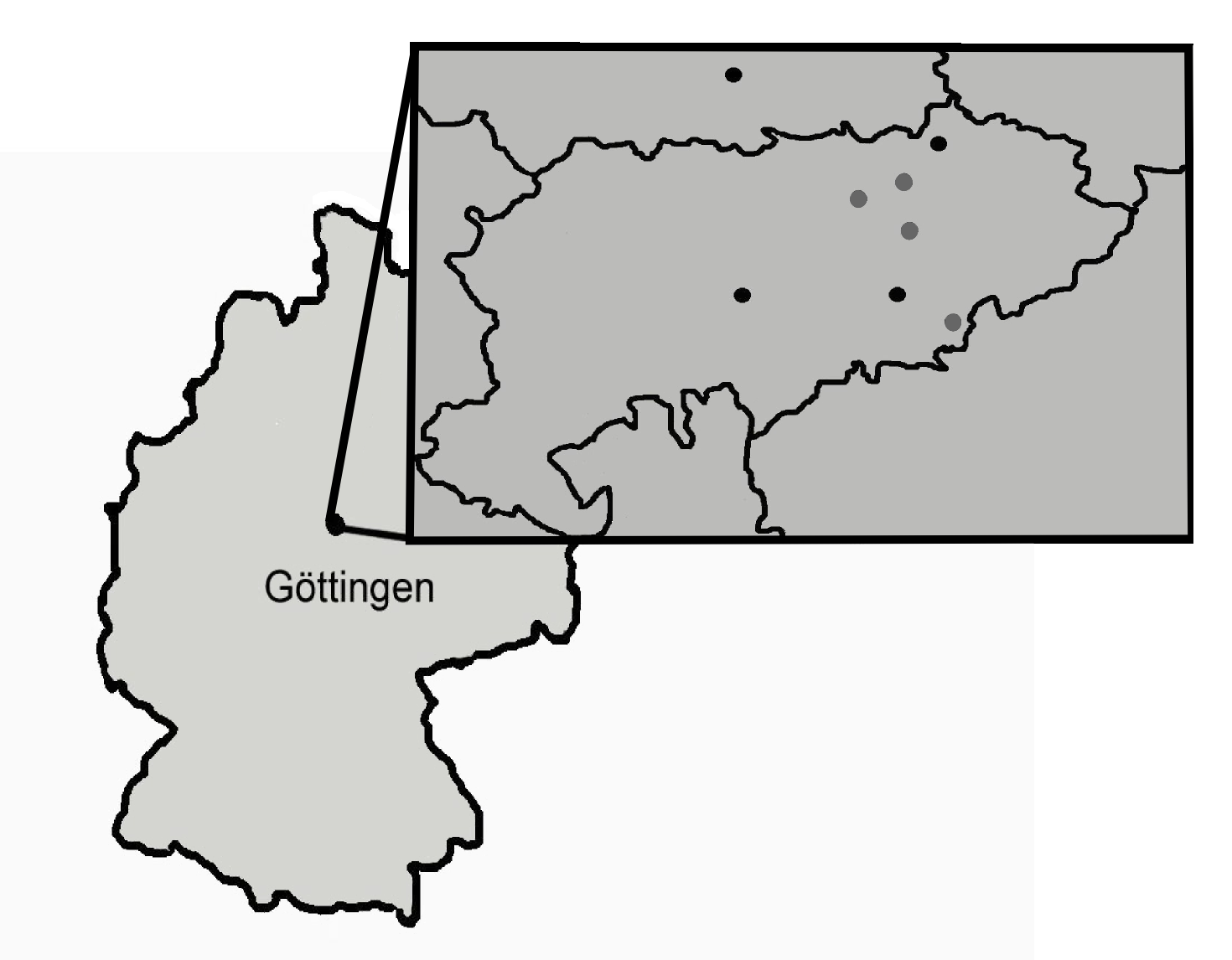

Supplement: Supplementary file 5 — Supplementary material 5 (TIFF 5556 kb) [file 442_2012_2366_MOESM5_ESM.tif]
